# Topics of Public Interest

**Published:** 1913-08

**Authors:** 


### TOPICS OF PUBLIC INTEREST.

---

A society of centenarians has recently been formed in Tokio, Japan, with a membership of 500 at the first meeting. The oldest member is a woman, aged 113.

Rosa Rutelli walked from Parma to a hospital in Milan, June 7, for relief for her first sickness, complaining of loss of appetite. She is a little over 102.

Asa Brainerd, 100 years old, a Civil War veteran, appeared before a committee of the Buffalo Aldermen, June 5, to protest against the erection of a cinemetograph theater.

Mrs. Elizabeth Maugherman died in June, aged 108. She had had seventeen children, four of whom survive her.

Rev. John Fryer Mesick, a graduate of Rutgers, 1834, the oldest living college man in the country, received the degree of LL. D. from his alma mater at the latest commencement.

Emma Wagner, the oldest woman in Arkansas, aged 112, recently walked six miles in three hours, without bad effect.

David Briggs of Westfield, N. Y., was 100 years old April 18.

Samuel Keefer of Dresden, near Penn Yan, has lately taken up scientific gardening as a study and recreation. He is over 100.

Adrian San Roman of the Province of Leon, Spain, died June 8, aged 114. He is survived by one son, aged 88, three grandchildren, aged 58, 59 and 63 respectively; thirteen great grandchildren, forty-five great great grandchildren, one great great great grandchild. Sixty-two direct descendants and 240 relatives attended his funeral.

Dr. Cho Choy of China, lately a resident of Cuba, died at Ellis Island at his immigration into this country, after a sudden illness of less than a day, at the reputed age of 150.

---

Dr. John Huston Finley, President of the College of the city of New York, has been elected by the State Board of Regents for an indefinite term as State Commissioner of Education, at a salary of $10,000. This is an admirable appointment.

---

The State Board of Regents, made the following appointments July 2:

State Board of Dental Examiners, William C. Deane, first district, and Albert M. Wright, third district, reappointed.

State Board of Pharmacy, George C. Diekman, New York, and Byron M. Hyde, Rochester, reappointed, and Thomas F. Raymow, Brooklyn, to succeed Clarence O. Bigelow, New York.

State Board of Examiners of Certified Public Accountants, Charles S. McCulloh and Samuel D. Patterson, New York.

State Board of Examiners in Optometry, Charles F. Prentice, New York, and George R. Fox, Buffalo.

State Board of Nurse Examiners, Miss Jane E. Hitchcock, New York, reappointed.

State Board of Medical Examiners, Earl H. King, Saratoga Springs, to succeed Dr. Lee H. Smith.

Inspector in Pharmacy, Harry N. Butler, New York.

The following academies were admitted to the university:

Eastport Union School, Genoa Union School, Hamilton Institute for girls, Saint Patrick's School, Buffalo, and Saint Mary's School, Swormville.

Municipal Seizure of Ice Plants. During a recent strike the Board of Health of Cincinnati seized and began the operation of ice plants, by the authority of the Mayor, Henry T. Hunt, who declared that a public emergency existed. We understand that the dispute between employers and employees has since been settled. Having in mind the recent strike of street car employees in Buffalo, Jamestown and other places, it occurs to us that municipal and state authorities should lay down and inforce the general rule that no dispute of this nature shall be made an endurance test on the part of the public. No economic question short of those resulting in political revolution should justify violence or serious interference either with the health, or convenience of the public. Unfortunately, the only way to prevent violence, seems to be by application of the principle of similia similibus. If it became generally known that the first few men who resorted to violence would be killed, economic disputes would be settled by peaceable methods and not by the logic of force.

---

The North Tonawanda Municipal Hospital has been named the DeGraff Memorial Hospital, in recognition of the donation of $20,000. Work has been begun under the charge of the Mayor, Dr. John A. Rafter. It will have thirty rooms, will be constructed without expense to the public, but will be jointly supported by both Tonawandas. We understand that the Hospital will be public both to the laity and to the profession.

---

Venereal Diseases are required to be reported as contagious in New York, Utah, New Jersey, Washington, California and, recently, Indiana. We question very much whether the encroachment on the old established principle of privileged communications is justified, or whether the law can be inforced.

---

Minimum Charge For Electricity. We regret very much to announce that the Public Service Commission, while forcing a somewhat more reasonable scale of charges, has confirmed the minimum charge of $12 a year. There is this advantage gained to the small consumer, that a fluctuation of use does not require him to pay more than a dollar a month if his average does not exceed this amount. This rate applies to Buffalo, but will doubtless effect other cities indirectly.

---

Tuberculosis Camp For Niagara Falls. Land has been secured north of Park avenue, between Main street and the River. The camp is expected to be ready before August 1.

The Buffalo Association For the Relief and Control of Tuberculosis announces that there are three institutions ready for the treatment of the three groups of cases mentioned editorially in the July issue: For incipient and early, non-infectious cases, the J. N. Adam Memorial Hospital at Perrysburg (of, for and by but not in) Erie County; for more advanced cases, the Open Air Camp on Grider street, near Kensington avenue; for advanced, bed-ridden cases, the remodeled Municipal Hospital on E. Ferry street, between Fillmore and Kehr. The first and last receive patients on application to the Buffalo Dept. of Health. The camp is under direct charge of the Association; there is a physician in attendance, but cases need not be relinquished. Patients will be cared for, night and day, throughout the pleasant weather, or may attend during the day only. For all classes of cases needing assistance, provision will be made.

---

Wood Alcohol Blindness. The Buffalo Association for the Prevention of Blindness has recently published in the daily press a warning against this poison, citing both immediately fatal and blinding cases. A Niagara Falls judge, in sentencing a man who had become drunk on Scotch whiskey, said: "If you want a strong drink, take wood alcohol. It is not nearly so bad as Scotch." Sarcasm of this sort is dangerous.

---

Eye Injuries From Golf Balls. The Massachusetts State Board of Health has recently investigated two cases, and has had analyses made of the liquid cores of a number of balls. Three types of solution have been found: Water, zinc chlorid solution, soft soap with talc or red lead in suspension.

---

### Statistics of Animals Killed For Food, 1912

1. Chicago, 12,910,506

2. Kansas City, 5,646,161.

3. South Omaha, 4,609,655

4. New York City, 3,034,685

5. East St. Louis, 2,966,292

6. South St. Joseph, 2,671,443

7. Boston, 1,826,044

8. Indianapolis, 1,598,503

9. Sioux City, 1,520,607

10. Buffalo, 1,381,271

232,687 whole carcasses and 494,328 parts were condemned, as well as nearly 18,000,000 pounds of prepared meat and its products.

---

### Automobile Notes

To prevent moisture and rain from obscuring the view, rub a little of a mixture of equal parts of kerosene and glycerin over the glass.

Do not pay more than 18 cents for gasolene nor more than 30 cents for engine oil, per gallon, unless you prefer to be extravagant.

There is no more efficient agent for promoting the sale of tires than a well oiled road with holes filled with small particles of corniferous limestone.

It needs no argument that an automobile going forty miles an hour in a city is a source of danger. Here are a few other dangers just as great: Searchlights used in the city; in-bound automobilists from clubs, with their own tanks too full; street commissioners who allow holes to develop so that vehicles pursue serpentine courses; children playing in the street and "jay-walkers"; drivers asleep on wagons; slowly moving vehicles in the middle of the street; the driver of any kind of a vehicle who, without reference to traffic rules, does not develop a sense of what the average careful driver must or would naturally do, in an emergency. It is this instinct of driving that renders rapid transit possible in the narrow, crowded streets of European cities, and it is the lack of it that causes accidents with no technical violation of any traffic law.

---

HEALTH SCHOOL FOR ROCHESTER. At the petition of the Woman's Union, the Rochester School Department is considering the establishment of courses in Dietetics, Physiology, Prophylaxis, Nursing, etc. It is probable that lectures will be given in the morning, afternoon and evening. This is a most important movement and one that will be imitated, we trust, by other cities.

